# Non-Coeliac Wheat Sensitivity: Symptoms in Search of a Mechanism, or a Distinct Well-Defined Clinical Entity? A Narrative Review

**DOI:** 10.3390/ijms262211174

**Published:** 2025-11-19

**Authors:** Stiliano Maimaris, Chiara Scarcella, Giusi Aurora Memoli, Carlotta Crisciotti, Annalisa Schiepatti, Federico Biagi

**Affiliations:** 1 Department of Internal Medicine and Therapeutics, University of Pavia, 27100 Pavia, Italy; giusiaurora.memoli01@universitadipavia.it (G.A.M.); carlotta.crisciotti01@universitadipavia.it (C.C.); annalisa.schiepatti01@universitadipavia.it (A.S.); federico.biagi@icsmaugeri.it (F.B.); 2 Istituti Clinici Scientifici Maugeri IRCCS, Gastroenterology Unit of Pavia Institute, Via Salvatore Maugeri 10, 27100 Pavia, Italy; chiara.scarcella@icsmaugeri.it

**Keywords:** non-coeliac gluten sensitivity, non-coeliac wheat sensitivity, wheat, gluten, gluten-free-diet

## Abstract

Non-coeliac wheat sensitivity (NCWS) is characterised by gastrointestinal and extra-intestinal symptoms following gluten/wheat ingestion in individuals without coeliac disease or wheat allergy but remains controversial due to symptom overlap with irritable bowel syndrome (IBS). This narrative review aims to provide a comprehensive, critical analysis of NCWS as a clinical and biological entity, examining the evidence for its distinction from related disorders. While self-reported rates are high (often >10%) in the general population, rigorous double-blind, placebo-controlled challenge (DBPCC) studies confirm the diagnosis in only a minority of cases (typically <30%). The clinical presentation is heterogeneous, combining IBS-like symptoms with systemic complaints such as “brain fog,” headaches, and fatigue. The pathophysiology is distinct from coeliac disease, involving innate immune activation, altered intestinal barrier function, and gut dysbiosis. Non-gluten wheat components, particularly fructans and amylase-trypsin inhibitors, are implicated as potential triggers. Diagnosis is challenging, requiring the exclusion of other disorders and adherence to complex dietary challenge protocols such as the Salerno Experts’ Criteria, which are impractical for routine clinical use. The search for validated biomarkers is a key research area and investigated candidates include serological markers such as IgG anti-gliadin antibodies, inflammatory markers such as faecal calprotectin, and proteins related to intestinal permeability such as zonulin, but results have been conflicting and require further validation. Management primarily involves elimination of wheat and gluten from the diet, although a low-FODMAP diet has also proven effective as an adjunctive treatment. In conclusion, NCWS is a clinical entity whose study and management are critically hampered by the absence of validated diagnostic criteria and biomarkers. Progress requires methodologically rigorous DBPCC trials to elucidate its mechanisms and develop reliable diagnostic tools.

## 1. Introduction

The term non-coeliac gluten sensitivity was first used in the 1970s to describe patients reporting functional diarrhoea related to gluten ingestion in the absence of coeliac disease (CD) [1,2] but was reintroduced more recently in the early 2010s to describe patients self-reporting a wide range of gastrointestinal and extra-intestinal symptoms apparently related to gluten ingestion [3,4,5]. It was later recognised that other components of wheat besides gluten may also play a role in the condition, leading to an update of the terminology to “non-coeliac wheat sensitivity” (NCWS) [6,7,8]. This shift was heavily influenced by key studies, for instance a rigorous double-blind, placebo-controlled crossover trial which found that gluten did not induce symptoms in individuals with self-reported gluten sensitivity once dietary fermentable oligo-, di-, mono-saccharides and polyols (FODMAPs), such as fructans, were reduced [6]. Further support came from research identifying other wheat proteins, such as amylase-trypsin inhibitors (ATIs), as potential activators of innate immunity [8], strengthening the case for NCWS being a more accurate term for the condition to better reflect the role of other components of wheat in its pathogenesis [7].

NCWS is a clinical syndrome characterised by heterogeneous gastrointestinal and extra-intestinal symptoms occurring after the ingestion of gluten and wheat-containing foods [9,10,11]. It is a diagnosis of exclusion that can only be made after both CD and wheat allergy (WA) have been ruled out [9,12,13,14,15]. The elimination of wheat/gluten from the diet typically results in improvement of symptoms and recurrence of symptoms if they are reintroduced in the diet [9,13].

However, NCWS remains a controversial and poorly defined condition due to many overlapping clinical features with irritable bowel syndrome (IBS) [16,17], lack of disease-specific biomarkers, and unclear pathogenetic mechanisms [9,11]. The lack of disease-specific biomarkers makes diagnosing NCWS especially challenging, with current approaches relying on excluding other alternative causes of symptoms followed by controlled dietary challenge protocols, particularly the Salerno Experts’ Criteria [12], which are very cumbersome to apply in everyday clinical practice.

Despite increasing clinical recognition of NCWS and growing research interest, significant gaps remain in our understanding of this condition. Considering recent advances in understanding the molecular mechanisms of NCWS, including the roles of innate immunity, intestinal barrier function, microbiome alterations, and the ongoing debate regarding the role of gluten versus other wheat components, a comprehensive critical evaluation of the current evidence is needed. This narrative review aims to provide an integrated and comprehensive overview of the epidemiology, pathophysiology, clinical features, biomarker research, diagnosis, and management of NCWS while critically reviewing the evidence supporting NCWS as a distinct clinical entity from related disorders such as CD, WA, and IBS.

## 2. Methods

**Figure 1:**
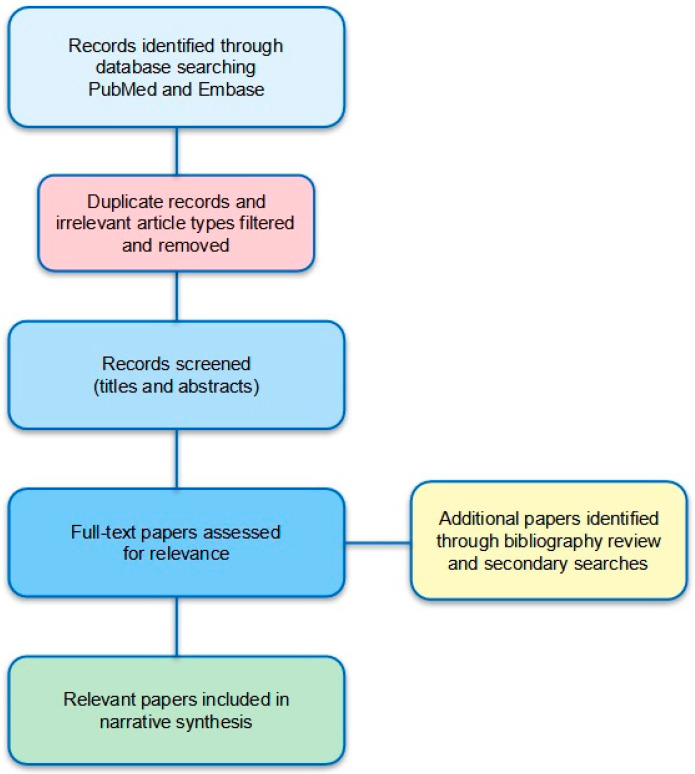
Flowchart illustrating literature search and study selection process.

A literature search on PubMed and Embase was conducted for relevant English-language full-text papers on non-coeliac gluten-wheat sensitivity from database inception to April 2025 using keywords for non-coeliac gluten sensitivity and wheat sensitivity. Figure 1 shows a flowchart of our literature review and paper selection process. We considered for inclusion peer-reviewed articles on NCWS and related topics in the English language. Case reports and conference abstracts were excluded. Duplicate citations and irrelevant article types were filtered and removed. Titles and abstracts of identified records were then screened by three reviewers (GAM, CC, and CS) and full texts of potentially relevant articles were then reviewed. The bibliographies of selected reviews and papers were also reviewed to identify other relevant papers that may have been missed by the literature search. Secondary searches by reviewers to identify additional relevant studies were also conducted throughout the review process. A narrative synthesis approach was chosen for our review rather than a systematic review methodology due to the broad aim of our review and the large heterogeneity in diagnostic methodologies and criteria across studies.

### 2.1. Epidemiology

The epidemiology of NCWS is difficult to define due to lack of disease-specific biomarkers, heterogeneous clinical manifestations, and reliance on complex symptom-based gluten challenge diagnostic protocols, which often confirm the diagnosis in only a minority of patients [18,19,20,21,22]. For these reasons, the epidemiology of NCWS is poorly defined. Due to these limitations, the majority of epidemiological studies have therefore focused on self-reported gluten sensitivity, generally reporting very high prevalence estimates in the general population, with most finding a female predominance. For instance, Australian survey data from 2015 and 2018 found a self-reported prevalence of NCWS of 13.8–13.9% among the Australian general population, with an annual incidence of 1.8% [23]. Self-reported data from a UK population-based study gave a similar figure of 13% [24]. Italian data from high school students found a similar self-reported prevalence of 12.2%, with 2.9% also following a gluten-free diet (GFD), despite only 23% of self-reporters having sought medical attention and only 14% having undergone serological testing for CD [21]. A more recent Italian survey estimated the overall prevalence at 6.4% [25].

Surveys from several other countries have reported slightly lower prevalence figures, although they were still very high. A survey in the Netherlands found 6.2% of respondents self-reported gluten sensitivity [26]. Similarly, a US online survey estimated the prevalence at 5.1% [27]. Data from South American countries varies but appears to be generally lower, with estimates of 7.6% in Argentina [28], 5.2% in Paraguay, [29], 3.1% in El Salvador [30], and 2.33% in Brazil [31].

However, self-reported prevalence figures significantly overestimate actual cases, as the majority of individuals who believe they are gluten-sensitive do not meet the criteria for NCWS. In this regard, a meta-analysis of 11 double-blind placebo-controlled gluten challenge studies found a pooled relapse rate of only 30% after gluten ingestion in patients self-reporting gluten sensitivity, with results between studies also showing extreme variability (7–77%) [32]. It has also been shown that many patients self-reporting gluten sensitivity cannot reliably distinguish gluten from a placebo, suggesting other factors such as FODMAP intolerance, nocebo, and other functional gastrointestinal disorders such as IBS may be responsible for symptoms in a large subset of patients [33,34,35,36]. It is also difficult to reconcile the data from many studies relying on self-reported data showing a prevalence close to 10% in the general population, with an estimated prevalence of only 6% among patients seen at the Center for Celiac Research at the University of Maryland between 2004 and 2010, a research centre with a clear interest in this condition [4].

NCWS may also rarely affect children. Data from an Italian multicentre, randomised, double-blind, placebo-controlled crossover (DBPCC) trial found that NCWS affects <1% of children with chronic gastrointestinal symptoms unrelated to CD or WA [20]. Among children with symptoms apparently related to gluten ingestion, NCWS was ultimately ruled out in >60% of cases [20], similarly to data from adult cohorts [11,32].

In conclusion, most surveys find a self-reported NCWS prevalence rate of close to 10% in the general population but, considering the results of double-blind placebo-controlled gluten challenge studies, ultimately it is likely only a minority of these individuals have NCWS.

#### Factors Contributing to Geographic and Temporal Variation in Prevalence

The substantial variation in self-reported NCWS prevalence across different geographic regions and studies (ranging from 2.33% in Brazil [31] to 13.9% in Australia [23]) warrants critical analysis of contributing factors. Several key elements may explain these disparities:

Dietary patterns and wheat consumption vary significantly across populations. Countries with higher wheat-based dietary traditions (such as Italy, Australia, and Northern Europe) show higher self-reported prevalence rates, possibly reflecting both greater exposure to the trigger and increased awareness of wheat-related symptoms [21,23,24]. Conversely, lower prevalence in some Latin American countries [30,31] may partly reflect different staple grain consumption patterns.

Survey methodology substantially impacts reported prevalence. Online surveys and social media-based recruitment [27] tend to yield higher prevalence estimates compared to population-based epidemiological studies, likely due to selection bias whereby symptomatic individuals are more likely to participate. Clinical studies from specialised centres report lower prevalence [4,20] but represent a pre-selected population already under medical investigation. The Italian high school student survey [21] is particularly revealing: while 12.2% self-reported NCWS, only 23% of them had sought medical attention and only 14% of them had undergone serological testing for CD, highlighting the disconnect between self-diagnosis and medical evaluation.

Healthcare access, awareness, and cultural factors also influence reporting. Regions with greater public awareness of gluten-related disorders and easier access to specialised gastroenterology services show higher diagnosis rates. Furthermore, cultural attitudes toward medically unexplained symptoms, acceptance of dietary modification as a health intervention, and the commercial availability of gluten-free products may all influence both symptom reporting and prevalence estimates.

Genetic background differences across populations may contribute to true prevalence variation. However, the lack of validated diagnostic biomarkers makes it impossible to determine whether geographic differences reflect true biological variation or primarily methodological and cultural factors.

Temporal trends also merit consideration. The dramatic increase in gluten-free diet adoption and NCWS awareness over the past decade [23,27] may have led to increased symptom attribution to wheat/gluten, potentially amplified by social media and wellness culture influences. This temporal effect likely contributes to the notably high prevalence in recent surveys compared to earlier epidemiological studies.

### 2.2. Clinical Presentation

Table 1 summarises the clinical manifestations that have been reported in patients with NCWS. These include a wide range of gastrointestinal and extra-intestinal symptoms that occur after gluten/wheat ingestion. Abdominal pain, bloating, diarrhoea, constipation, and flatulence are among the most common gastrointestinal complaints, making it very difficult to distinguish NCWS from IBS based solely on the clinical picture [16,17,37,38].

Data suggest that a subset of patients affected by functional gastrointestinal disorders such as IBS may have underlying NCWS. A UK study found that 42.4% of IBS patients self-reported gluten sensitivity [51], similarly to a Korean study that found 33.6% of IBS patients reported NCWS-like symptoms [52]. A double-blind placebo-controlled gluten challenge study found that 46.1% of IBS patients pre-treated with a low-FODMAP GFD experienced worsening of symptoms with gluten challenge [34]. A trial involving patients with refractory functional dyspepsia found that 35% experienced improvement on a GFD, although only 18.5% relapsed after gluten reintroduction [39], suggesting a subset of patients with functional dyspepsia have underlying NCWS.

Extra-intestinal symptoms are also frequently reported in NCWS and can significantly impact quality of life. These are very heterogeneous and include non-specific symptoms such as fatigue, musculoskeletal pain, and headaches. Neurological complaints are common, including migraine-like headaches, cognitive disturbances (‘brain fog’), ataxia, and peripheral neuropathy [40,41,42,43]. A UK study comparing neurological manifestations in patients with CD and NCWS found they were similar in both groups and responded equally well to a GFD [40]. One retrospective study even observed clinical improvement of chronic low back pain and spondyloarthritis-like features after following a GFD in 79% of patients [53]. Interestingly, some data suggest that gluten exposure may also influence mood independent of gastrointestinal symptoms. In one randomised cross-over trial including 22 apparently gluten-sensitive IBS patients, participants reported significantly more depressive symptoms during gluten challenge than with a placebo despite finding no difference in terms of gastrointestinal symptoms between gluten and the placebo [45]. Conversely, another study reported significantly greater worsening of abdominal and non-abdominal symptoms after gluten challenge in NCWS patients than in CD patients but found no significant differences regarding level of somatization, quality of life, anxiety, or depressive symptoms [46]. Anaemia has also been reported to occur significantly more frequently in NCWS than in IBS patients (34.8% vs. 17.4%, *p* = 0.03), though significantly less frequently than in coeliac patients [44].

### 2.3. Associated Conditions

Although specific serological markers of immune activation are lacking in NCWS, evidence supports a role of innate immune activation in NCWS. Several autoimmune conditions have been reported to be associated with NCWS, including Hashimoto’s thyroiditis and various rheumatologic disorders [54]. An association of NCWS with fibromyalgia has also been reported [55], although the data is in contrast with other data suggesting that the prevalence of NCWS in patients with fibromyalgia does not differ from that in the general population [56]. Cutaneous manifestations morphologically similar to eczema, psoriasis, or dermatitis herpetiformis which are responsive to a GFD have also been reported in NCWS patients [47]. An increased prevalence of nickel allergies and contact dermatitis has also been reported in NCWS compared to IBS patients, with cutaneous symptoms being reported more frequently after gluten ingestion [49]. Bone health is also a concern, with osteopenia or osteoporosis affecting up to 40% of NCWS patients, often in the context of low BMI and insufficient calcium intake [50]. Women with NCWS have also been reported to have a higher prevalence of gynaecological symptoms compared to IBS patients, with improvement in gynaecological symptoms such as recurrent vaginitis, cystitis, and menstrual irregularities after wheat elimination [48].

### 2.4. Pathophysiology

NCWS is pathophysiologically distinct from both WA and CD, which are well-characterised immune-mediated disorders. The mechanisms underlying NCWS are considered to be complex and heterogeneous and remain poorly understood [17,57]. Hypothesised pathogenetic mechanisms include the innate immune response to wheat components, increased intestinal permeability, gut microbiota alterations, and gut–brain axis interactions. However, lack of disease-specific biomarkers or clear endoscopic and/or histological findings and substantial clinical overlap with other functional disorders such as IBS have made delineating the pathogenetic mechanisms of NCWS particularly challenging [14,57]. Table 2 summarises the main hypothesised pathophysiological mechanisms.

#### 2.4.1. Experimental Models and In Vitro Evidence

In vitro and experimental models have been crucial for investigating the potential pathogenic pathways of NCWS, as human studies are often confounded by placebo effects, compounding the difficulty in isolating specific dietary triggers. At a cellular level, gliadin has been shown to induce oxidative and endoplasmic reticulum (ER) stress in intestinal cell lines and mouse models, which can contribute to inflammation and tissue damage [70,71]. The gut microbiota is also implicated, with studies showing that ATIs can promote a state of dysbiosis that worsens inflammatory conditions like colitis in mice [72].

Beyond gluten, ATIs have been identified as potent activators of the innate immune system via toll-like receptor 4 (TLR4) in myeloid cells [73], though this has been recently challenged by findings suggesting that co-purified lipopolysaccharide (LPS) may be responsible for this effect [74]. Regardless, ATIs have been demonstrated to act as adjuvants, exacerbating pre-existing allergic inflammation in both the gut and airways in mouse models [75,76].

Finally, animal models have provided direct links between wheat-related immune responses and extra-intestinal pathology. For instance, anti-gliadin immunity has been shown to trigger CNS inflammation and blood–brain barrier breakdown in mice [77], providing plausible mechanisms for the diverse systemic symptoms reported in NCWS patients.

#### 2.4.2. Immune Mechanisms

In contrast to CD, which is characterised by a well-defined adaptive immune response to gluten showcased by the strong HLA-DQ2/DQ8 association, T-cell activation and production of tissue transglutaminase antibodies [78], it is the innate immune response that is hypothesised to play a greater role in NCWS [58,79]. Activation of toll-like receptor 4 by components such as wheat amylase-trypsin inhibitors (ATIs) has been hypothesised to induce production of pro-inflammatory cytokines, such as TNF-α and IL-8 [58,59,60]. This activation occurs in myeloid cells such as monocytes, macrophages, and dendritic cells. Upon binding to the TLR4-MD2-CD14 receptor complex, ATIs are thought to trigger intracellular signalling cascades, primarily through the MyD88-dependent pathway. This culminates in the activation of transcription factors, most notably nuclear factor-kappa B (NF-kB), which orchestrates the transcription of a wide array of pro-inflammatory genes, including those for TNF-α, IL-1β, IL-6, and IL-8 [58,59,60]. Systemic immune activation against microbial and wheat components with evidence of intestinal epithelial damage has also been hypothesised to play a role [8].

Although lacking the specific adaptive response characteristic of CD, some evidence also suggests a mild involvement of the adaptive immune system. NCWS patients may test positive for anti-gliadin antibodies (AGAs), non-specific antibodies that tend to normalise quickly after the initiation of a GFD, often within months. A recent study evaluated the use of AGAs as a screening tool for NCWS in a large sample of IBS patients, identifying 12.4% as potentially gluten-sensitive [37]. After 6 weeks of GFD, 87% of the AGA-positive patients showed clinical improvement, and all responders experienced a recurrence of symptoms with gluten reintroduction. Serum AGA levels were also significantly reduced in responders, suggesting a potential role as a biomarker in clinical practice [37]. An increase in IFN-γ mRNA in duodenal biopsies observed in NCWS patients after gluten exposure also supports a limited activation of Th1 immunity [68]. Self-reported NCWS patients also show increased expression of cytokines associated with Th1 and Th17 compared to healthy controls but to a lesser extent than in coeliac patients [69].

#### 2.4.3. Gut Barrier Function and Microbial Translocation

Increased intestinal permeability (“leaky gut”) has been proposed as one of the potential pathogenetic mechanisms of NCWS. This is hypothesised to facilitate the passage of microbes and antigens across the intestinal epithelium, contributing to the activation of the innate immune system [8]. Zonulin, a regulatory protein of intestinal tight junctions, is among the most studied. Upon binding to its receptors it triggers intracellular signalling that leads to the disassembly of key tight junction proteins, particularly zonula occludens-1 and occludin. This process increases intestinal permeability and not only allows the translocation of dietary antigens such as gluten peptides and ATIs but also microbial components such as LPS, which can further amplify the inflammatory response by engaging TLR4 on lamina propria immune cells. In particular, Barbaro et al. reported significantly higher serum zonulin levels in NCWS patients compared to IBS-D patients and asymptomatic controls, similar to those observed in active CD [61]. In an ex vivo study on duodenal biopsies conducted by Hollon et al., gliadin exposure increased intestinal permeability in all examined groups (active CD, CD in remission, GS, and healthy controls), but with a more pronounced response in active CD and in subjects with NCWS, suggesting a common role of gluten in modulating the intestinal barrier [62]. However, a study by Talley et al. found no difference in serum zonulin between NCWS and IBS or functional dyspepsia patients [80]. Similarly, another study evaluating markers of epithelial barrier function and bacterial translocation in self-reported gluten-sensitivity individuals with IBS found no effect of gluten ingestion on these markers [81].

#### 2.4.4. Gut Microbiota

Gut dysbiosis is increasingly recognised as playing a role in various gastrointestinal disorders, including IBS and coeliac disease, and has also been hypothesised in NCWS [60,82]. Comparative studies between CD, NCWS, IBS, and healthy subjects have highlighted both overlaps and specificities in microbial profiles.

In a study by Naseri et al., CD patients showed a higher presence of *Firmicutes*, while those with NCWS exhibited a lower abundance of *Bacteroidetes* [63]. In both groups, as well as in IBS patients, a reduction in *Bifidobacterium* spp. was observed compared to healthy controls. The *Firmicutes*/*Bacteroidetes* ratio was altered in both CD and NCWS. Multivariate analysis further showed that the microbial profiles of IBS and NCWS were more similar to those of healthy controls than to those of coeliac patients [63]. A study by Ponzo et al. found that gluten-sensitive subjects exhibited lower levels of *Bacteroides* and *Parabacteroides* and higher levels of *Blautia* and *Streptococcus* compared to subjects responsive to the placebo [64].

Dieterich et al. also observed significant microbial differences between NCWS patients and controls, with variations linked to both the low-FODMAP diet and the GFD [65]. In this regard, a study comparing low-gliadin bread to a GFD showed a potentially beneficial change in the microbiota of NCWS patients, with an increase in butyrate-producing bacteria such as *Roseburia* and *Faecalibacterium* [83]. However, one study did not detect significant changes in the faecal microbiota (by 16S analysis or metagenomics) in response to a 14-day gluten challenge in patients with CD or NCWS already on a GFD [84]. The intestinal mycobiota (fungal microbiota) has so far been little explored. In this regard, the study by Ponzo et al. did not detect any significant differences in its composition between subjects with suspected NCWS in response to gluten or a placebo [64].

Further confirmation of the association between the intestinal microbiota and NCWS comes from a study conducted in Mexico, which analysed the duodenal and faecal microbiota in subjects with NCWS, CD, and healthy controls. In NCWS patients, a greater abundance of the genus *Actinobacillus* was observed at the duodenal level and of the family *Ruminococcaceae* at the faecal level. After four weeks of GFD, an increase in the genus Pseudomonas was detected in the duodenum, suggesting a selective effect of the diet on specific microbial components correlated with symptomatology [85].

In recent years, interest has also expanded towards the modulatory role of the microbiota in the response to gluten. In a randomised, crossover, double-blind, placebo-controlled study, short-term exposure to gluten, fructans, and placebo in subjects with self-reported NCWS showed that fructans provoke a greater number of gastrointestinal symptoms compared to gluten [86] and are potentially key mediators of the complex interplay of factors in NCWS. However, no significant variations were found in overall microbial diversity, fermentation metabolites (SCFA), or faecal levels of NGAL/LCN2. Only the bacterial group *Eubacterium coprostanoligenes* was reduced after fructan ingestion, in association with worsening symptoms. Furthermore, the baseline microbial composition was found to correlate with the individual response to different dietary stimuli, suggesting a possible predictive role of personal bacterial profiles [86].

The functional consequences of microbiota alterations in NCWS may be mediated through microbial metabolites that interact with host cells via specific molecular receptors and signalling pathways. SCFAs, primarily butyrate, propionate, and acetate, are produced through bacterial fermentation of dietary fibres and resistant starches in the colon. Key SCFA-producing genera include *Faecalibacterium prausnitzii*, *Roseburia* spp., and *Eubacterium* spp., several of which have been found to be reduced in NCWS patients [83,86].

SCFAs exert multiple beneficial effects on intestinal homeostasis through distinct molecular mechanisms. Butyrate serves as the primary energy source for colonocytes and regulates intestinal barrier function by enhancing expression of tight junction proteins including claudin-1, occludin, and zonula occludens-1 through histone deacetylase inhibition and activation of AMP-activated protein kinase (AMPK) signalling [83]. Additionally, SCFAs bind to G protein-coupled receptors (GPR) on colonocytes and immune cells: GPR41 (FFAR3), GPR43 (FFAR2), and GPR109A. Activation of these receptors on intestinal epithelial cells promotes mucin production and antimicrobial peptide secretion, while activation on immune cells (particularly regulatory T cells and macrophages) induces anti-inflammatory responses through NLRP3 inflammasome inhibition and reduced NF-kB activation [60,82].

The reduction in SCFA-producing bacteria observed in some NCWS patients [83] could theoretically impair barrier integrity and promote low-grade inflammation, contributing to symptom generation. However, direct measurements of faecal SCFA concentrations in NCWS patients have yielded inconsistent results, with some studies finding no significant differences compared to controls [86], suggesting that SCFA dysregulation may only occur in a subset of NCWS patients or that other metabolic pathways are more relevant.

Secondary bile acids, produced by bacterial 7α-dehydroxylation of primary bile acids, may also potentially play a role in NCWS patients with prominent gastrointestinal symptoms by influencing intestinal motility, fluid secretion, and the immune response. However, bile acid profiles have not been systematically characterised in NCWS populations [60,82].

#### 2.4.5. Genetic Factors

Unlike CD, which is strongly associated with HLA-DQ2 and HLA-DQ8 haplotypes (present in over 95% of patients), only approximately 50% of NCWS patients possess one of these haplotypes, a frequency that is substantially higher than that in IBS controls (30%), according to an Italian study, which is similar to that in the general population [41] but not close to that in CD. It is interesting to note that one study detected a reduction in serum zonulin levels only in HLA-DQ2/8-positive NCWS patients after wheat elimination, suggesting a possible interaction between HLA predisposition and intestinal permeability [61].

A study by Gambino et al. examined the distribution of immunoglobulin killer receptors (KIR) and highlighted significant differences between NCWS patients, coeliac subjects, and healthy controls. NCWS patients showed a lower frequency of several inhibitory and activating KIR genes (KIR2DL1, -2DL3, -2DL5, -2DS2, -2DS3, -2DS4, -2DS5, and -3DS1) and a higher frequency of -3DL1. Some alleles, such as KIR2DL5, -2DS4, and -2DS5, have been suggested as independent protective predictors against the development of NCWS [87].

Interest in the molecular mechanisms underlying NCWS has also spurred analyses of gene expression in intestinal biopsies. These studies have identified differential gene expression profiles (DEGs) and miRNAs (including non-coding RNAs) in the duodenal mucosa [88] and potentially also in peripheral blood leukocytes of NCWS patients compared to controls [89]. In a subsequent study, comparison with CD DEGs showed a significant overlap (~30%), especially in long non-coding RNAs, suggesting a potential common origin that diverges toward different inflammatory phenotypes [90].

Finally, an evolutionary hypothesis has been proposed by Sazzini et al., who identified balanced selection on divergent haplotypes of the CXCL10/CXCL11 genes, which are involved in the CXCR3 inflammatory axis. In particular, the H1 haplotype, which promotes a greater inflammatory response, was found to be twice as frequent in terms of homozygosity in NCWS patients compared to healthy controls [91].

#### 2.4.6. Neuro-Immune Interactions

Some authors suggest a possible involvement of the enteric nervous system in NCWS. A study by Giancola et al. investigated the interactions between mast cells and nerve structures in the duodenal submucosa. Although no differences in neuronal density were found among groups, the percentage of mast cells in close proximity to nerves (within 15 µm) was significantly increased in patients with NCWS, CD, and functional dyspepsia compared to healthy controls [67]. In NCWS subjects, mast cell density was positively correlated with the severity of pain and bloating, while the close proximity between mast cells and nerve fibres was associated with a greater number of gastrointestinal symptoms, particularly bloating and pain [67]. This close anatomical relationship suggests a functional bidirectional communication. Upon activation, mast cells degranulate, releasing a host of potent neuroactive mediators, including histamine, serotonin, and proteases (such as tryptase). These mediators can directly act on adjacent enteric nerve endings. For example, histamine can activate H1 receptors on nociceptive (pain-sensing) afferent neurons, while tryptase can activate protease-activated receptor 2 (PAR-2) on submucosal neurons. This activation lowers the firing threshold of these neurons, leading to visceral hypersensitivity—an abnormal state where normal physiological stimuli, such as gut distension from gas, are perceived as painful. This mechanism provides a direct cellular and molecular link between an immune response and the characteristic IBS-like symptoms of pain and bloating in NCWS [67].

#### 2.4.7. The Role of Gluten, Placebo, and Nocebo

Table 3 summarises the key findings regarding placebo and nocebo effects in NCWS revealed by gluten challenge studies. Although evidence suggests other components of wheat besides gluten, such as fructans, might be responsible for, or contribute to, symptoms in NCWS patients [60,92,93], gluten remains the most investigated component. Some double-blind placebo-controlled challenge (DBPCC) studies have shown symptom recurrence in a subset of patients after gluten exposure compared to placebos [22,32]. However, results are inconsistent and several rigorous DBPCC studies failed to show a specific effect of gluten over placebos [6,33,94]. Zanini et al. found only 34% of patients meeting clinical criteria for NCWS correctly identified gluten-containing flour compared to gluten-free flour [35] and Dale et al. found only 20% correctly identified gluten, with the group overall reporting worse symptoms on the placebo [33]. An interesting crossover DBPCC including 61 patients with self-reported gluten sensitivity by Di Sabatino et al. found that although patients had significantly greater symptoms with gluten than the placebo (*p* = 0.03), when considering individual patients, the majority had similar symptom scores with gluten and the placebo; after using a statistical cut-off of >2 standard deviations in symptom scores between gluten and the placebo to identify individuals with “true NCGS”, only three patients met this criterion [95]. Finally, a meta-analysis of 11 gluten re-challenge studies showed no significant difference in symptom relapse risk between gluten and placebo challenges, although when restricting the analysis to studies employing the Salerno criteria, a significant difference emerged [32].

Further complicating matters, the amount of gluten tolerated by patients may vary. Roncoroni et al. found some NCWS patients reacted to relatively low gluten doses (3.5 g/day), while others tolerated higher doses (8–13 g/day) [97]. A growing body of evidence indicates that psychological factors and patient expectations may significantly influence symptom perception. The significant impact of the nocebo effect (symptoms triggered by expecting gluten, even when consuming placebo) complicates challenge studies. An elegant study by De Graaf et al. showed that the expectation alone of eating gluten significantly increased symptoms, and that expectation had a larger impact on increasing symptoms than actually consuming gluten, although the combination of both expectation and actual gluten intake showed the largest increase in symptoms, so an additional effect of gluten beyond nocebo could not be entirely ruled out [36]. A scoping review by An et al. analysed 16 DBPCC studies, finding that 8 reported a greater symptomatic response to gluten than controls, but highlighted significant methodological limitations and inconsistencies across studies (participant selection, challenge protocols, and reporting), concluding that the evidence for gluten’s pathogenetic role was inconsistent [96]. Further highlighting the role of psychological factors, another study found that compared to coeliac patients, individuals with NCWS were significantly more likely to doubt vaccine safety (41.3% vs. 26.4%), decline vaccination (30.9% vs. 24.2%), avoid genetically modified foods, consume only organic products, distrust the Food and Drug Administration (FDA) as a source of information, and believe that a GFD improves energy and concentration [98].

#### 2.4.8. FODMAPs (Fermentable Oligo-, Di-, Monosaccharides, and Polyols)

FODMAPs are poorly absorbed carbohydrates that are fermented by gut bacteria, producing gas and causing osmotic effects, leading to symptoms like bloating, pain, and diarrhoea in susceptible individuals. It is noteworthy that wheat is a major source of fructans, a type of FODMAP. Skodje et al. conducted a rigorous DBPCC crossover trial in 59 individuals with self-reported gluten-sensitivity, finding that fructan challenge (2.1 g/day) induced significantly higher overall symptom scores and bloating scores compared to both gluten (5.7 g/day) and placebo challenges [66], suggesting fructans, rather than gluten, may be the primary trigger in many self-diagnosed NCGS individuals. An earlier study by Biesiekierski et al. found that after being placed on a low-FODMAP diet, patients self-reporting gluten sensitivity found no effect on symptoms from gluten challenge [6]. Corroborating this, Ajamian et al. showed that reducing FODMAP intake significantly reduced symptoms and reversed apparent colonic epithelial injury (lower syndecan-1) in IBS patients with self-reported NCWS, regardless of gluten challenge [81]. However, a study by Barone et al. found that in IBS patients subjected to a low-FODMAP-GFD run-in phase followed by a subsequent gluten challenge, 19.2–46.1% (depending on criteria) were gluten-sensitive, suggesting both gluten and FODMAP intolerance may play a role [34]. In line with this, Dieterich et al. found that both a low-FODMAP diet and a GFD improved symptoms in NCWS patients, with GFD also reducing duodenal IELs and goblet cells [65].

#### 2.4.9. Other Wheat Factors

ATIs are non-gluten proteins found in wheat that are resistant to digestion and have been proposed as likely triggers for NCWS [58] due to in vitro data suggesting they may activate the innate immune system via toll-like receptor 4 [59,73]. However, currently no human challenge studies specifically assessing ATIs as NCWS triggers have been conducted [96]. Thus, while ATIs triggering an immune response are a plausible candidate based on in vitro data, direct evidence linking them to symptoms in NCWS is currently lacking.

Other potential triggers that have been proposed include modern wheat processing (refining, vital gluten addition, and intensive kneading), as it might increase indigestible fractions or alter gluten structure, contributing to NCWS [99]. The potential tolerability of ‘ancient’ grains was explored by two studies. Ianiro et al. found lower symptom scores with Senatore Cappelli durum wheat compared to standard wheat in NCWS patients in a double-blind crossover trial [100], while Seidita et al. noted that NCWS may tolerate ‘ancient’ grain varieties (including Timilia/Tumminia, Perciasacchi, Khorasan, Senatore Cappelli, and Russello) better and that a subset of patients continue to consume them post-diagnosis with milder or no symptoms compared to modern wheat [101]. Potential explanations for better tolerability of ‘ancient’ grains may include differences in ATI content, variations in fructan levels, differences in processing methods, and differences in gluten protein content and composition across wheat strains. However, further studies are needed to support these findings and to better elucidate potential underlying mechanisms for the better tolerability of these ‘ancient’ grain varieties in NCWS.

### 2.5. Challenges in Diagnosis and Differential Diagnosis

Diagnosing NCWS remains a significant challenge due to the lack of specific biomarkers and the overlap of symptoms with CD and IBS [12,14,102]. Therefore, the first essential step is to exclude CD and WA [12,14,103]. Exclusion of CD requires negative coeliac-specific serology (IgA tissue transglutaminase/endomysial antibodies) and, if seronegative CD is suspected, a duodenal biopsy showing normal villous architecture (Marsh 0-I) while the patient is consuming gluten [78,104]. However, patients often present to medical professionals while already on a GFD, making it difficult to confirm or rule out CD and usually a gluten-challenge followed by coeliac serology and duodenal biopsy is necessary [105,106]. HLA-DQ2/DQ8 testing may be useful in this situation as absence of DQ2/DQ8 rules out CD [78,104]. WA is typically excluded based on clinical history and negative specific IgE tests or skin prick tests for wheat allergens [103]. However, non-IgE-mediated WA is harder to exclude [103]. Table 4 summarises the key differences between NCWS, CD, WA, and IBS.

#### 2.5.1. The Role and Limitations of Gluten Challenge

Currently, the “gold standard” for diagnosis of NCWS, particularly in research settings, involves a DBPCC protocol, often following the Salerno Experts’ Criteria [12]. This involves first documenting symptom improvement on a GFD and then performing a DBPCC with crossover, typically involving periods of gluten administration vs. placebo, separated by washout. Diagnosis requires a significant (>30%) symptom increase on gluten compared to placebo, measured using validated scales [12]. This process is impractical, time-consuming, expensive, and extremely difficult to implement in routine clinical practice. Moreover, compliance with this diagnostic protocol can be an issue and the high frequency of placebo/nocebo responses significantly complicates interpretation. A meta-analysis by Lionetti et al. highlighted the low overall confirmation rate and similarity between gluten and placebo responses at DBPCC, although adherence to the Salerno criteria improved discrimination [32]. Further complicating matters, the optimal gluten vehicle and placebo for DBPCC also remain debated [115].

#### 2.5.2. Distinguishing NCWS from IBS

The symptomatic overlap between NCWS and IBS is substantial, leading to diagnostic confusion [16,17]. Many patients diagnosed with IBS, particularly IBS-D or IBS-M, also report symptom triggers related to food, including wheat [17]. Some studies suggest a significant proportion of IBS patients may also have underlying NCWS [38,52,116]. In this regard, Shahbazkhani et al. found 25.7% of IBS patients responsive to GFD relapsed specifically on gluten challenge [38] while Barmeyer et al. found 34% of IBS-D/M patients responded long-term to a GFD, suggesting underlying NCWS [116].

However, clinical features are insufficient for differentiation between IBS and NCWS. Although extra-intestinal symptoms are common in IBS, some studies suggest NCWS patients might have a higher frequency of extra-intestinal symptoms, associated atopy/allergies, or certain non-specific laboratory findings (e.g., IgG AGA, eosinophilia) compared to IBS patients [41,108]. DBPCC remains the most reliable way to confirm gluten/wheat as the specific trigger for symptoms in IBS patients but is extremely impractical as a diagnostic tool for routine clinical practice, and the potential confounding role of FODMAPs makes this even more complex to assess [34,60].

#### 2.5.3. Histological Findings

While CD is characterised by villous atrophy, crypt hyperplasia, and an increased intraepithelial lymphocyte count [117], NCWS typically shows normal or near-normal duodenal histology. Several studies report subtle inflammatory histological changes in NCWS duodenal biopsies compared to controls. Reported findings include a slightly increased IEL density [68,111], an uneven/clustered IEL distribution pattern [105], and increased eosinophil counts in the duodenum [108,113,114]. Similarly, increased eosinophil counts have also been found in the rectal mucosa of NCWS patients [113] and, compared to IBS controls, an expansion of IFN-gamma-producing type 1 innate lymphoid cells has also been detected in the rectal mucosa of NCWS patients, with a subsequent decrease after patients were placed on a wheat-free diet [118]. However, these differences can be detected only after statistically analysing data from many patients and cannot be relied upon to make a diagnosis in individual patients.

### 2.6. Biomarkers

The search for reliable biomarkers for NCWS is a major research focus but none are currently validated for clinical use [79,102]. Research on biomarkers for NCWS has ranged from serological markers, inflammatory molecules, genetic parameters, immunophenotypic markers, and metabolomic profiles, but current evidence remains inconclusive. Table 5 summarises the biomarkers and diagnostic tools that have been investigated for NCWS.

By definition, the antibodies used for the diagnosis of CD—anti-tissue transglutaminase (tTG), anti-endomysium (EMA), and anti-deamidated gliadin peptides (DGP)—as well as wheat-specific IgE, are negative in NCWS patients [14,78,104]. IgG AGA can be present in about 50% of patients with suspected NCWS, but their sensitivity and specificity is low [108,109,110] and they are not sufficient to support or confirm the diagnosis [110]. Moreover, AGAs tend to normalise quickly in NCWS patients, often disappearing within months after initiating a GFD [119].

The role of zonulin is controversial, with some data suggesting that elevated serum levels may distinguish NCWS from IBS-D, in combination with clinical data [61]. However, other data suggests that zonulin is not able to effectively discriminate between NCWS, IBS, or functional dyspepsia [80]. Other markers related to barrier integrity and intestinal inflammation (I-FABP, claudin-1, LBP, and sCD14) have been investigated but have not shown significant differences between IBS patients, healthy controls, and suspected NCWS patients on a GFD [120].

Analysis of serum cytokines has yielded some preliminary results, finding some differences in cytokine profiles (IL-6, IL-8, IL-15, and IFN-gamma) between CD, NCWS, and healthy controls [121,122]. However, although scientifically interesting, these findings require validation on larger cohorts and are currently not useful from a diagnostic point of view.

A pilot study using nuclear magnetic resonance spectroscopy suggested that the serum metabolomic and lipoprotein profiles (mainly related to HDL cholesterol) could distinguish CD (and potential CD) from NCWS with good accuracy (AUC 0.90 and 0.83, respectively), and hypothesised that these differences may be due to alterations in the microbiota of NCWS patients [125].

Flow cytometry applied to intraepithelial lymphocytes (IELs) has provided some interesting results. A study by Martín-Cardona et al. found TCRγδ+ IEL percentage was able to distinguish CD patients from NCWS with good accuracy (accuracy of 0.88 using a cut-off of >13.31% TCRγδ+ IELs), even in patients already on a GFD, suggesting a possible role in excluding CD in patients presenting already on a GFD [124]. One pilot study investigated the ALCAT 5 test for NCWS and found 64% concordance with the results of a DBPCC, suggesting potential utility as a preliminary screening tool to select patients for more in-depth investigations such as DBPCC, although clinical applicability is currently limited due lack of validation and relatively low diagnostic accuracy [126].

Faecal calprotectin (FC) is an established marker of intestinal inflammation commonly used in the differential diagnosis between functional disorders and organic pathologies, such as chronic inflammatory bowel diseases (IBD). In a multicentric study, Seidita et al. evaluated FC levels in NCWS patients confirmed via a DBPCC, comparing them to patients with IBS and functional dyspepsia. The results showed elevated FC levels (defined as >41 µg/g) in 31.3% of NCWS patients, compared to negativity in all IBS or functional dyspepsia patients (specificity 98%, sensitivity 58.6%, and AUC 0.755) [123], and FC levels also significantly decreased on a wheat-free diet, normalising in 65.1% of the positive patients after ≥6 months [123]. However, although interesting, these results still require validation and further study before FC can be used to support a diagnosis of NCWS.

### 2.7. Management

Table 6 summarises current treatments for NCWS. Currently, the main treatment for NCWS remains the elimination of wheat and gluten from the diet, with most patients reporting significant symptom improvement afterwards, although response rates vary substantially across studies [18]. Regarding the long-term effects of a GFD in NCWS, although symptoms generally improve after starting a GFD, studies have found that many patients continue to experience mild persistent symptoms, inadequate nutrient intake, and poor health-related quality of life [127,128]. FODMAPs, particularly fructans, have also been implicated in the pathogenesis of NCWS, and a low-FODMAP diet may provide additional symptom improvement in NCWS patients according to the results of several studies [6,34,65,81]. Some limited data also suggest that ‘ancient’ wheat varieties may be better tolerated by NCWS patients [100,101].

Several studies have examined adherence to a GFD in NCWS, but the data are inconsistent. One study found that adherence to a GFD was good, particularly in patients showing a good clinical response [116]. Other studies found similar [132] to slightly lower adherence to a GFD in patients with NCWS compared to coeliac patients [133]. However, NCWS patients were more likely to be self-educated about GFDs [132], and lower adherence in NCWS was associated with poorer quality of life [133].

Beyond dietary interventions, several other treatments have been evaluated but evidence to support their use is currently weak. An Italian non-randomised, open-label, parallel-group pilot study reported that combining probiotics (Bifidobacterium longum ES1) with a GFD led to greater reductions in both intestinal and extra-intestinal symptoms and improved stool consistency compared to diet alone [129]. Oral enzyme supplements designed to digest gluten have also been evaluated but the evidence is mixed. One study evaluated an enzyme mixture containing Aspergillus-derived peptidases and proteases and found significantly improved overall symptoms during a gluten challenge [130]. However, another study found that a proline-specific endopeptidase (P1016) did not improve symptoms compared to placebo during gradual gluten reintroduction [131].

Data on long-term outcomes in patients with NCWS are lacking. In particular, it should be noted that while CD is well-known to be associated with an increased mortality compared to the general population due to the development of malignant complications [134], there is currently no evidence suggesting poor long-term outcomes in NCWS. Therefore, while a strict GFD is of fundamental importance in CD for histological recovery of villous atrophy and preventing complications [78,104], its role in NCWS is mainly for management of symptoms, so strict adherence to a GFD does not appear to be necessary for patients with NCWS based on current data. Moreover, the levels of gluten required in NCWS to trigger symptoms has been shown to vary significantly between individuals (3.5–13 g/day) [97], suggesting that dietary strictness should be individualised in NCWS. Furthermore, while cross-contamination needs to be carefully avoided in CD, considering the low doses involved, this is not likely to be a significant issue for patients with NCWS.

## 3. Limitations

Although our review has provided a comprehensive overview of the evidence underpinning NCWS as a distinct entity, this review has several limitations that should be acknowledged. Firstly, as summarised in Table 7, in many cases the level of evidence was low, often based on only retrospective observational data, or in some cases on only pre-clinical studies, with high quality evidence mostly being limited to the investigation of dietary triggers of NCWS and the role of placebos and nocebos. Secondly, as a narrative review, we did not employ the systematic methodology of a systematic review, such as a rigorous predefined study selection strategy or a formal quality assessment of the included studies. Our approach is susceptible to some degree of selection bias and reflects the authors’ expert interpretation of the field rather than an exhaustive, quantitative synthesis. The literature on NCWS is characterised by significant heterogeneity in study design, diagnostic criteria, and patient populations, which further complicates the formation of definitive conclusions. A substantial geographic bias exists in the available literature, with the majority of high-quality studies originating from Europe, with limited representation from Asia, Africa, and Latin America. This geographic concentration may limit the generalizability of findings and represents an important gap in the literature. Finally, the ongoing debate and lack of validated biomarkers for NCWS mean that the very definition of the condition can also vary significantly between studies, a fundamental challenge that this review reflects.

## 4. Conclusions

Although NCWS is increasingly recognised as a distinct clinical and biological entity that is characterised by both IBS-like gastrointestinal symptoms and diverse extra-intestinal symptoms apparently triggered by gluten and wheat ingestion, significant controversy and uncertainty remain. The greatest hurdle to research, diagnosis, and management of NCWS remains the lack of validated disease-specific biomarkers and reliance on complex dietary challenge diagnostic protocols that are complicated by significant placebo and nocebo effects. For the clinician, currently NCWS should be considered a diagnosis of exclusion after ruling out coeliac disease and wheat allergy. Diagnosis and management rely on an empirical trial of a GFD, but the key role of fructans means a low-FODMAP diet is also a valid or adjunctive therapeutic option. Crucially, unlike in coeliac disease, the goal is symptom control, so dietary strictness can be individualised and minor cross-contamination is unlikely to be clinically significant. The key challenge remains the identification of specific reliable disease biomarkers to move beyond cumbersome DBPCC and the high placebo/nocebo response rate. Future research should focus on the identification and study of potential biomarkers in vitro and in vivo, followed by large-scale high-quality DBPCC trials employing standardised protocols, validated outcome measures, and carefully designed challenge materials including both gluten and other dietary triggers to improve the reliability of diagnostic biomarkers.

## Figures and Tables

**Table 1:** Clinical manifestations reported in non-coeliac wheat sensitivity.

Category	Symptoms
Gastrointestinal	Abdominal pain, bloating, altered bowel habits (diarrhoea/constipation), flatulence, dyspepsia [16,17,37,38,39]. Symptoms often overlap with irritable bowel syndrome.
Extra-intestinal/Systemic	Fatigue, lethargy (‘brain fog’), headaches (often migraine-like), musculoskeletal pain (fibromyalgia-like), anaemia [40,41,42,43,44].
Neurological/Psychiatric	Cognitive disturbances, peripheral neuropathy, ataxia, anxiety, depression [40,41,42,43,45,46].
Dermatological	Eczema-like or psoriasis-like skin rashes, dermatitis herpetiformis-like lesions [47].
Other Associations	Gynaecological issues (menstrual irregularities, recurrent vaginitis) [48], increased prevalence of atopy and nickel allergy [49], reduced bone mineral density (osteopenia/osteoporosis) [50].

**Table 2:** Hypothesised pathophysiological mechanisms in non-coeliac wheat sensitivity.

Mechanism	Hypothesised Effect	Key Evidence/Mediators
Innate Immune Activation	Wheat components trigger an innate immune response in the gut mucosa, leading to low-grade inflammation.	Amylase-trypsin inhibitors activating toll-like receptor 4 [58,59]; increased pro-inflammatory cytokines (e.g., TNF-α, IL-8) [58,60].
Increased Intestinal Permeability	A compromised gut barrier (“leaky gut”) allows microbial products and dietary antigens to cross into circulation, causing immune activation.	Increased serum zonulin in some studies [61]; ex vivo studies showing increased permeability upon gliadin exposure [62].
Gut Microbiota Dysbiosis	Alterations in the composition and function of the gut microbiome contribute to symptoms and inflammation.	Altered *Firmicutes*/*Bacteroidetes* ratio; reduced *Bifidobacterium*; changes in microbial profiles vs. controls and CD patients [63,64,65].
FODMAP Intolerance	Fermentation of short-chain carbohydrates (especially fructans from wheat) by gut bacteria leads to gas, bloating, and pain.	DBPCC trials showing fructans induce more symptoms than gluten in some self-reported NCWS individuals [6,66].
Neuro-immune Interactions	Increased interaction between immune cells (mast cells) and the enteric nervous system enhances visceral hypersensitivity.	Increased mast cell density in close proximity to submucosal nerve fibres, correlating with pain and bloating severity [67].
Mild Adaptive Immunity	A low-level adaptive immune response, distinct from and less intense than that seen in CD.	Presence of IgG-AGA in some patients [37]; slight increase in Th1/Th17-related cytokines and duodenal IFN-γ mRNA [68,69].

FODMAP: fermentable oligo-, di-, monosaccharides, and polyols; DBPCC: double-blind placebo-controlled challenge; NCWS: non-coeliac wheat sensitivity; AGA: anti-gliadin antibodies; TNF: Tumour Necrosis Factor; IL: Interleukin.

**Table 3:** Key findings regarding placebo and nocebo effects revealed by gluten challenge studies.

Challenge	Key Findings	Implications for Diagnosis and Research
High Placebo and Nocebo Effects	A significant portion of patients report symptom recurrence when given a placebo they believe contains gluten.	Complicates interpretation of DBPCCs; highlights the strong psychological component and symptom anticipation [33,35].
Power of Expectation	The mere expectation of consuming gluten can cause a greater increase in symptoms than covertly consuming gluten itself.	The nocebo effect may be a greater driver of symptoms than gluten in many individuals, clouding diagnostic clarity [36].
Inability to Distinguish	In several blinded trials, patients as a group could not reliably distinguish gluten from placebo, sometimes reporting worse symptoms on placebos.	Questions the specificity of gluten as the sole trigger in a large subset of self-diagnosed NCWS patients [33].
Inconsistent DBPCC Results	Meta-analyses show high variability in gluten relapse rates (7–77%) and often no significant difference from the placebo unless strict criteria are used.	Standardisation of DBPCC protocols (e.g., Salerno Criteria) is critical to obtain reliable and comparable results [32].
Methodological Variation	Wide variation in participant selection, gluten/placebo vehicle, challenge dose, and outcome measures across studies.	Lack of standardisation hinders the ability to compare studies and draw firm conclusions on gluten’s pathogenetic role [96].

DBPCC: double-blind placebo-controlled challenge; NCWS: non-coeliac wheat sensitivity.

**Table 4:** Key differences between of NCWS, coeliac disease, wheat allergy, and irritable bowel syndrome.

Feature	Non-Coeliac Wheat Sensitivity	Coeliac Disease	Wheat Allergy	Irritable Bowel Syndrome
Pathophysiology	Innate immunity [58,79], barrier dysfunction [61,62], microbial dysbiosis suspected [60].	HLA-DQ2/DQ8 associated adaptive T-cell response to gluten [78,104].	IgE-mediated or non-IgE-mediated reaction to wheat proteins [103].	Visceral hypersensitivity, altered motility, gut–brain axis dysfunction [107].
Key Symptoms	IBS-like GI symptoms + diverse extra-intestinal (fatigue, ‘brain fog’, headache, pain) [16,17,38,40,41,42,43].	GI symptoms (diarrhoea, malabsorption) + extra-intestinal [78,104].	Rapid onset (mins–hrs) urticaria, angioedema, asthma; or delayed GI symptoms [103].	Recurrent abdominal pain related to defecation, change in stool frequency/form [107].
Symptom Onset	Hours to days after ingestion [9,13].	Variable, can be delayed [78,104].	IgE-mediated: minutes to 2 h. Non-IgE: hours to days [103].	Chronic, fluctuating; can be triggered by food (incl. wheat), stress [107].
Serology	Negative CD/WA serology. IgG AGA may be positive (~50%) [108,109,110].	Positive anti-tTG, anti-EMA, anti-DGP IgA [78,104].	Positive wheat-specific IgE (for IgE-mediated WA) [103].	Negative for specific markers [107].
Genetics (HLA)	HLA-DQ2/DQ8 in ~50% (vs ~30% in general population) [41].	Strong association with HLA-DQ2/DQ8 (>95%) [78,104].	No specific HLA association [103].	No specific HLA association [107].
Histology (Duodenum)	Normal or minimal changes (Marsh 0-I), mild IEL increase, eosinophils [68,108,111,112,113,114].	Villous atrophy, crypt hyperplasia, significant IEL increase (Marsh III) [78,104].	Usually normal [103].	Usually normal [107].
Diagnosis	Exclusion of CD/WA, followed by DBPCC (Salerno criteria) [12].	Positive serology + duodenal biopsy showing villous atrophy [78,104].	Clinical history + positive IgE tests or skin prick tests [103].	Symptom-based criteria (Rome IV) after excluding organic disease [107].
Primary Trigger	Gluten, fructans, ATIs, other wheat components [32,58,66].	Gluten [78,104].	Wheat proteins (gluten and others) [103].	Various foods (FODMAPs), stress, visceral sensitivity [107].

AGA: anti-gliadin antibody; ATIs: amylase-trypsin inhibitors; CD: coeliac disease; DBPCC: double-blind: placebo-controlled challenge; DGP: deamidated gliadin peptides; EMA: endomysial antibodies; FODMAP: fermentable oligo-, di-, monosaccharides, and polyols; GI: gastrointestinal; HLA: human leukocyte antigen; IBS: irritable bowel syndrome; IgA: Immunoglobulin A; IgE: Immunoglobulin E; IEL: intraepithelial lymphocytes; NCWS: non-coeliac wheat sensitivity; tTG: tissue transglutaminase antibodies; WA: wheat allergy.

**Table 5:** Potential biomarkers and diagnostic tools for non-coeliac wheat sensitivity.

Biomarker	Proposed Role	Key Findings and Limitations
IgG Anti-Gliadin Antibodies	Serological marker of gluten exposure/reaction.	Positive in ~50% of NCWS, but low sensitivity and specificity [108,109,110]. Not diagnostic. Tend to normalise on GFD [119].
Zonulin	Marker of intestinal permeability.	Controversial. Some studies show higher levels vs. IBS [61], others find no difference [62]. Not reliable for diagnosis.
I-FABP, LBP, sCD14	Markers of epithelial barrier integrity and microbial translocation.	No significant differences found between NCWS, IBS, and healthy controls in most studies [81,120].
Serum Cytokines (e.g., IL-6, IL-8)	Markers of systemic inflammation.	Some studies show different profiles vs. CD/controls, but findings are inconsistent and not validated for diagnostic use [121,122].
Faecal Calprotectin (FC)	Marker of intestinal inflammation.	One study found elevated FC in ~31% of NCWS patients (vs. 0% in IBS) [123], suggesting potential but requiring validation.
Duodenal Histology (IELs, Eosinophils)	Quantifying subtle mucosal inflammation.	Slight increases in IELs and eosinophils reported, but overlap with controls is large [68,108,111,112,113,114]. Not useful for individual diagnosis.
Flow Cytometry (TCRγδ + IELs)	Differentiating CD from NCWS in patients on GFD.	Promising results in one study to exclude CD (even on GFD), but not a positive marker for NCWS [124]. Needs validation.
Metabolomics/Lipoprotein Profiles	Identifying unique metabolic signatures.	Pilot study suggested potential to distinguish NCWS from CD, but still preliminary and requires validation [125].
ALCAT 5 Test	In vitro food sensitivity testing.	One pilot study showed 64% concordance with DBPCC. Not validated and has low accuracy for clinical use [126].
Genetic Markers (KIR, CXCL10/11)	Identifying genetic predisposition.	Research findings of associations exist [85,89], but these are not diagnostic markers for clinical practice.

CD: coeliac disease; CXCL: C-X-C Motif Chemokine Ligand; DBPCC: double-blind placebo-controlled challenge; FC: faecal calprotectin; GFD: gluten-free diet; IELs: intraepithelial lymphocytes; IBS: irritable bowel syndrome; I-FABP: intestinal fatty acid-binding protein; IL: Interleukin; KIR: killer-cell immunoglobulin-like receptor; LBP: lipopolysaccharide-binding protein; NCWS: non-coeliac wheat sensitivity; sCD14: Soluble CD14; TCR: T-cell receptor.

**Table 6:** Treatment strategies for non-coeliac wheat sensitivity.

Intervention	Description	State of Evidence
Gluten-Free Diet (GFD)	Strict elimination of wheat, barley, rye, and related grains.	**Primary Treatment.** Most patients improve, but response rates vary and mild symptoms may persist [18,127,128].
Low-FODMAP Diet	Restriction of fermentable oligosaccharides, disaccharides, monosaccharides, and polyols.	**Evidence for Symptom Control.** Effective, especially as wheat is a major source of fructans (a FODMAP) [6,34,65,81]. May be combined with GFD.
‘Ancient’ Wheat Grains	Consumption of older wheat varieties (e.g., Senatore Cappelli, Khorasan).	**Limited Evidence.** Some studies suggest better tolerability in a subset of patients, but not a substitute for GFD in sensitive individuals [100,101].
Probiotics	Supplementation with specific bacterial strains (*Bifidobacterium longum* ES1).	**Preliminary Evidence.** One open-label pilot study showed improved symptoms when combined with a GFD [129]. More research needed.
Enzyme Supplements	Oral enzymes designed to break down gluten peptides in the gut.	**Mixed/Weak Evidence.** Some small studies show modest benefit [130], while others show no effect compared to placebo [131]. Not a validated treatment.

FODMAP: fermentable oligo-, di-, monosaccharides, and polyols; GFD: gluten-free diet.

**Table 7:** Summary of key findings and approximate level of evidence in NCWS research.

Domain	Finding	Primary Study Types	Level of Evidence *
**Epidemiology**	High prevalence of self-reported wheat sensitivity (~5–15%).	Cross-sectional surveys.	**Low**
	Only a minority (<30%) of self-reported cases are confirmed by DBPCC.	Meta-analysis of DBPCCs, individual DBPCCs.	**High**
**Clinical Presentation**	Extensive symptom overlap with IBS.	DBPCCs in IBS cohorts, reviews, comparative studies.	**Moderate**
	Extra-intestinal symptoms (e.g., fatigue, “brain fog”, headache) are common.	Observational studies (retrospective, cross-sectional), case series.	**Low**
**Pathogenesis: Triggers, Placebo, Nocebo**	The nocebo/placebo effect is a major confounder in symptom reporting.	Rigorous DBPCCs designed to assess expectancy effects, meta-analyses.	**High**
	Fructans (a FODMAP in wheat) trigger more symptoms than gluten in many self-reported cases.	Rigorous DBPCC crossover trials.	**High**
**Pathogenesis: Biological Mechanisms**	Innate immune system activation is implicated.	In vitro cellular assays, animal models, ex vivo biopsy studies.	**Pre-clinical**
	Intestinal permeability (“leaky gut”) may be increased, but evidence is conflicting.	Observational studies measuring biomarkers (e.g., zonulin), ex vivo experiments.	**Low/Pre-clinical**
	Gut microbiota composition may be altered compared to controls.	Cross-sectional comparative 16S rRNA/metagenomic studies.	**Low**
**Diagnosis and Management**	No validated biomarkers currently exist for diagnosis.	Numerous biomarker discovery studies (observational) with conflicting results.	**Low**
	A gluten-free diet is the primary management for symptom control.	Observational studies, run-in phases of DBPCCs (indirect evidence).	**Moderate**
	A low-FODMAP diet is also effective for symptom control in many individuals.	DBPCCs, comparative dietary trials.	**High**

* High: Systematic reviews and meta-analyses; high-quality DBPCCs. Moderate: Smaller DBPCCs, prospective observational studies. Low: Retrospective studies, cross-sectional surveys, biomarker studies with conflicting results. Pre-clinical: In vitro, animal, or ex vivo studies. DBPCC: double-blind, placebo-controlled challenge; IBS: irritable bowel syndrome; FODMAP: fermentable oligo-, di-, monosaccharides, and polyols.

## Data Availability

No new data were created or analyzed in this study. Data sharing is not applicable to this article.

## Abbreviations

AGA	Anti-gliadin antibodies
AMPK	AMP-activated protein kinase
ATI	Amylase-trypsin inhibitors
AUC	Area Under the Curve
BMI	Body Mass Index
CD	Coeliac disease
DBPCC	Double-blind, placebo-controlled challenge
DEG	Differentially expressed gene
DGP	Deamidated gliadin peptide
EMA	Endomysial antibodies
ER	Endoplasmic reticulum
FC	Faecal calprotectin
FDA	Food and Drug Administration
FODMAP	Fermentable oligo-, di-, monosaccharides, and polyols
GFD	Gluten-free diet
GPR	G protein-coupled receptor
HLA	Human leukocyte antigen
IBD	Inflammatory bowel disease
IBS	Irritable bowel syndrome
I-FABP	Intestinal fatty acid-binding protein
IFN-γ	Interferon-gamma
IgG	Immunoglobulin G
IL	Interleukin
IEL	Intraepithelial lymphocyte
KIR	Killer-cell immunoglobulin-like receptor
LBP	Lipopolysaccharide-binding protein
LPS	Lipopolysaccharide
NCWS	Non-coeliac wheat sensitivity
NF-kB	Nuclear factor-kappa B
NLRP3	NLR Family Pyrin Domain Containing 3
PAR-2	Protease-activated receptor 2
sCD14	Soluble CD14
SCFA	Short-chain fatty acid
TCR	T-cell receptor
TLR4	Toll-like receptor 4
TNF	Tumour Necrosis Factor
tTG	Tissue transglutaminase antibodies
WA	Wheat allergy
